# Neonatologists’ decision-making for resuscitation and non-resuscitation of extremely preterm infants: ethical principles, challenges, and strategies—a qualitative study

**DOI:** 10.1186/s12910-021-00702-7

**Published:** 2021-09-25

**Authors:** Alice Cavolo, Bernadette Dierckx de Casterlé, Gunnar Naulaers, Chris Gastmans

**Affiliations:** 1 Centre for Biomedical Ethics and Law, KU Leuven, Kapucijnenvoer 35/3, 3000 Leuven, Belgium; 2 Academic Centre for Nursing and Midwifery, KU Leuven, Kapucijnenvoer 35/4, 3000 Leuven, Belgium; 3 Pregnancy, Fetus and Newborn, Department of Development and Regeneration, KU Leuven, UZ, Herestraat 49/7003 21, 3000 Leuven, Belgium

**Keywords:** Prematurity, Extremely preterm, Ethics, Decision-making, Resuscitation

## Abstract

**Background:**

Deciding whether to resuscitate extremely preterm infants (EPIs) is clinically and ethically problematic. The aim of the study was to understand neonatologists’ clinical–ethical decision-making for resuscitation of EPIs.

**Methods:**

We conducted a qualitative study in Belgium, following a constructivist account of the Grounded Theory. We conducted 20 in-depth, face-to-face, semi-structured interviews with neonatologists. Data analysis followed the qualitative analysis guide of Leuven.

**Results:**

The main principles guiding participants’ decision-making were EPIs’ best interest and respect for parents’ autonomy. Participants agreed that justice as resource allocation should not be considered in resuscitation decision-making. The main ethical challenge for participants was dealing with the conflict between EPIs’ best interest and respect for parents’ autonomy. This conflict was most prominent when parents and clinicians disagreed about births within the gray zone (24–25 weeks). Participants’ coping strategies included setting limits on extent of EPI care provided and rigidly following established guidelines. However, these strategies were not always feasible or successful. Although rare, these situations often led to long-lasting moral distress.

**Conclusions:**

Participants’ clinical–ethical reasoning for resuscitation of EPIs can be mainly characterized as an attempt to balance EPIs’ best interest and respect for parents’ autonomy. This approach could explain why neonatologists considered conflicts between these principles as their main ethical challenge and why lack of resolution increases the risk of moral distress. Therefore, more research is needed to better understand moral distress in EPI resuscitation decisions.

*Clinical Trial Registration*: The study received ethical approval from the ethics committee of UZ/KU Leuven (S62867). Confidentiality of personal information and anonymity was guaranteed in accordance with the General Data Protection Regulation of 25 May 2018.

## Introduction

The World Health Organization (WHO) defines extremely preterm infants (EPIs) as infants born before 28 completed weeks of gestation compared to the 40 weeks of a normal pregnancy [1]. EPIs typically need resuscitation at birth. Depending on the specific medical conditions, resuscitation can consist of different interventions of increasing intensity, such as ventilation and oxygenation, chest compressions, and administration of epinephrine and/or volume expanders [2, 3]. However, deciding whether to resuscitate EPIs can be difficult. In a recent meta-analysis, Myrhaug et al. reported survival rates in high-income countries as the following survival rates (mean 95% CI): 7.3% of all live births at gestational age (GA) 22 weeks; 25.7% at 23 weeks, 53.9% at 24 weeks, 74% at 25 weeks, around 80% at 26 weeks, and 90% at 27 weeks [4]. Moreover, EPIs have an increased risk of mild (e.g. behavioral disorders) to severe (e.g. neurosensory, motor, cognitive, and behavioral impairments) disability compared to term infants [5–7]. The risk of severe impairments is 36.3% for EPIs born at 22 weeks, 22.1% at 23 weeks, 19.1% at 24 weeks, 14% at 25 weeks, and 4% at 27 weeks [4]. Finally, other factors other than the GA contribute determining the individual probability of survival with good outcomes, e.g. clinical conditions at birth, administration of prenatal steroids, technological equipment and pharmaceuticals available at the hospital, and the occurrence of postnatal events (e.g. intracranial hemorrhage) [4, 6, 8]. These figures are in the same range for Flanders where recently the mortality and neurocognitive morbidity was reported [9]. Hence, despite having good statistical data at a population level, it is difficult to determine the specific survival probability of each individual EPI and whether their quality of life will be good [10].

Clinical uncertainty about prognosis raises key ethical questions. In a systematic review of the ethical literature, we found disagreements on whether GA is an appropriate criterion for deciding whether to resuscitate, showing once again that making EPI resuscitation decisions is difficult [11]. Authors also suggested that EPIs’ best interest and parents’ autonomy should be the main principles informing the decision-making [11]. However, the review’s description is merely theoretical. It does not shed light on whether neonatologists actually use these principles in real practice and, in case, how they use them to make EPI resuscitation decisions.

Empirical studies of neonatologists’ perspectives about EPI resuscitation focused mainly on their attitudes, i.e. whether they would resuscitate EPIs at different GAs [12–18]. The majority of these studies employed quantitative methodologies. These offered a good overview of *what* neonatologists prefer regarding EPI resuscitation but produced little insight into *how* neonatologists make these decisions and *how* they ethically legitimize their decisions. The few qualitative studies on the topic produced important insights on neonatologists’ preferences in terms of counselling [19, 20] and end of life decisions [21]. They also suggested that the main ethical neonatologists encounter relate to appropriate counselling, parental involvement, and dealing with clinical uncertainty [22–25]. However, many of these studies also included other healthcare professionals’ and parents’ views, making it difficult to discern the views of neonatologists [23–26]. Hence, a nuanced understanding of what ethical principles neonatologists balance in real-world decision-making and how they deal with ethical challenges is still lacking.

To address these gaps, we conducted a qualitative study in Belgium aimed at understanding neonatologists’ clinical–ethical decision-making for resuscitation at birth of EPIs. More specifically, we aimed at understanding what ethical principles neonatologists use, what ethical challenges they face, and how they use those principles to deal with the challenges.

## Methods

As we were interested in how Belgian neonatologists use ethical principles in their clinical–ethical decision-making regarding EPI resuscitation, we used a qualitative design supported by the Grounded Theory approach [27, 28]. We followed the Consolidated Criteria for Reporting Qualitative Research guidelines (COREQ) to report the results of the study [29].

### Setting

Belgium is divided in three independent regions: Flanders (population: 6,629,143); Wallonia (population: 3,645,243); and Brussels (population: 1,218,255) [30]. There are currently 19 NICUs active in Belgium employing 113 neonatologists. Flanders has an official guideline that provides advice on when to resuscitate EPIs delineated by GA [31]: From 26 weeks EPIs should always be resuscitated; under 24 weeks EPIs should not be resuscitated unless it is the explicit wish of the parents and after they are well informed; between 24 and 25 weeks (i.e. the grey zone) the decision-making is individualized. The decision between resuscitation and non-resuscitation is done case by case, looking at all the relevant factors (not GA alone), through shared decision-making between parents and healthcare providers. NICUs in Wallonia and Brussels have their own internal written or oral institutional recommendations. The majority of these latter NICUs guidelines, however, use the same thresholds as those delineated in the Flemish guideline.

### Recruitment

Participants were firstly recruited through the gatekeeper strategy [32]. The president of the Belgian Society of Neonatology invited members to participate in the study by sending them an information package containing a letter of invitation; an information brochure describing the study and the research team; an informed consent form; and a demographic questionnaire. Participants themselves were encouraged to invite other potentially interested colleagues (i.e. snowball method) [32].

Interested neonatologists returned the signed informed consent form and the completed questionnaire. The interviewer then contacted them to ask if they had study questions and to schedule an appointment.

Based on answers on the questionnaire we selected participants who (1) were practicing neonatologists in Belgium, (2) were involved in at least one decision-making for resuscitation at birth of EPIs, (3) were willing to participate, and (4) were willing to do the interview in English.

### Data collection

AC conducted 20 individual, semi-structured, face-to-face interviews between September 2019 and October 2020. The interviewer is a white, 30-year-old, nulligravida, PhD student with a background in philosophy and bioethics. Interviews took place at the participants’ hospitals.

We developed the interview guide based on previous literature reviews and a pilot interview [11, 12]. Throughout the data collection period, we further refined the guide based on the preliminary results of the interviews completed to date. To understand what the main ethical challenges are in real practice and how neonatologists deal with these challenges, we asked participants to describe real past cases in which they had to decide whether to resuscitate an EPI at birth. We then asked in-depth questions on that specific case and decision-making (e.g. how did he/she decide to resuscitate? why? What were the main issues? What was parents’ request? How did he/she discuss the case with parents?).

The interviews lasted on average one hour (range 37–82 min) and were conducted at the participants’ hospitals. The interviewer did not take field notes but all interviews were audiotaped and verbatim transcribed with the participants’ consent. Data collection ended at saturation.

### Data analysis

Consistent with the Grounded Theory, data analysis and data collection occurred simultaneously and interactively. Interviews were analyzed following the Qualitative Analysis Guide of Leuven (QUAGOL) [33, 34]. The QUAGOL is divided into two parts; each part is further subdivided into five steps.

In the first part, we coded the transcribed interviews using pen and paper. We analyzed interviews using the following five steps. (1) Interviews were read repeatedly to familiarize ourselves with the material. (2) For each interview, we developed a narrative scheme containing the key messages of the interview, using participants’ words. (3) We then developed individual conceptual schemes in which we highlighted the main themes emerging from each interview. This was the first step in which the concrete “raw data” were analyzed to extract qualitative concepts describing those data in more conceptual terms. (4) We re-read the interviews with their conceptual schemes in mind to check whether the scheme fit with the main messages of the interviews and whether we overlooked important information. The schemes were adjusted where necessary. (5) We developed a general conceptual scheme describing the common themes that emerged from all the interviews.

In the second part of QUAGOL, we continued coding the interviews by means of Nvivo12 software (QRS International, 2020), as described in the following five steps. (1) We used the general conceptual scheme from part 1 to create a list of codes in Nvivo 12. (2) Relevant fragments of each interview were digitally linked in the software to one or more codes. (3) The digitally linked fragments were then analyzed using systematic within-case and across-case analysis. (4) We organized and described concepts emerging from the analysis in a comprehensive general framework, which served as the basis to report the results of our study (5).

It is important to note that, although these analysis stages are described here as being carried out linearly, we constantly moved back-and-forth between analysis stages and phases, as prescribed in the QUAGOL approach [33, 34]. Finally, each stage of this analysis was carried out by an interdisciplinary team comprising the interviewer, a professional ethicist, and an expert in qualitative research. It is important to note that a neonatologist was involved in the study design, in the preparation of the interview guide, and in the revision of the results. However, he was not included in the analysis of the interviews because it would have been difficult to ensure the anonymity of participants considering that the Belgian neonatologists’ population is rather small and that the transcripts included in-depth descriptions of real cases.

### Ethics

The ethics committee of UZ/KU Leuven (S62867) approved the study. All participants received written and verbal information about the study. Their participation was voluntary, and the informed consent procedure was respected. All data were treated confidentially.

## Results

### Participants’ characteristics

Characteristics of participant neonatologists’ varied widely in age (range 34–63 years) and professional experience (range 2–30 years). Most of the participants were women (n = 15), and 15 of the 20 identified as Roman Catholic. At the time of the interviews, participants practiced in 10 NICUs with different bed capacities (range 15–40 beds). Other demographic and professional characteristics are presented in Table 1.

**Table 1:** Participants’ and NICUs’ demographic characteristics

	No.	%
*Participants’ characteristics*		
Sex		
Male	5	25
Female	15	75
Age, year range		
30–35	2	10
36–40	4	20
41–45	2	10
46–50	5	75
51–55	1	5
56–60	4	20
61–65	2	10
Religious affiliation		
Roman catholic	15	75
Liberal/no affiliation	5	25
Protestant, Muslim, Jewish, Other (specify)	0	0
Years of experience in NICUs (excluding years of training)		
1–5	3	15
6–10	4	20
11–15	2	10
16–20	3	15
21–25	4	20
26–30	3	15
> 30	1	5
Number of self-reported cases in the last year		
0	1	5
1	1	5
2	4	20
3	3	15
4	2	10
5 or more	9	45
*NICUs’ characteristics*		
Belgian region		
Flanders	5	50
Wallonia	3	30
Brussels	2	20
Hospital demographic		
Academic	5	50
Non-academic	5	50
Number of beds in the unit		
15–20	4	40
21–25	0	0
26–30	4	40
31–35	1	10
35–40	1	10

### Ethical principles

Based on participants’ accounts of past cases, we identified two main ethical principles guiding participants’ decision-making for resuscitation at birth of EPIs: EPIs’ best interest and respect for parents’ autonomy. Generally, participants’ ethical decision-making can be described as an attempt to balance these two principles. Interviewees told us that in the majority of cases balancing EPIs’ best interest and respect for parents’ autonomy was fast, easy, and almost unnoticeable. However, there were cases in which attempting to balance the principles was more complex. In these situations, participants essentially assigned the two principles different weights, depending on the EPI’s GA. Within the gray zone, clinical uncertainty is greatest, making it very difficult to determine whether resuscitation is truly in the EPI’s best interest. Hence, parents’ autonomy was valued more. Outside the gray zone, EPIs’ outcomes are more certain and, therefore, EPIs’ best interest was the main guiding principle.

However, participants’ ethical decision-making in the described cases was not solely reduced to balancing best interest and respect for autonomy. Interviewees appeared to have a more comprehensive approach to decision-making and considered other relevant principles. Because they viewed the EPI and the parents as a family unit, interviewees considered parents’ interests, their capacity to cope with either a dead or a severely disabled child, and how this could affect the EPI’s wellbeing. In doing so, they took into account justice implications. They reflected on how to take this holistic approach without discriminating against families. However, they only considered justice in terms of equality, not in terms of resource allocation. Such a comprehensive approach played a more prominent role when the balancing of EPIs’ best interest and parents’ autonomy was not straightforward. For quotes illustrating participants’ ethical principles, see Table 2.

**Table 2:** Illustrative quotes: Ethical principles

EPIs’ best interest	
	“We always say that we are the defender of the child so if there is a chance of good outcome we should give that chance to the child.” (PART. 4)
	“(*Referring to a case in which parents insisted on resuscitation and the baby died after months*) We had a long way with the infant and there was a lot of suffering with the infant and in the end he passed away. And then I wander whether this suffering is… what is the need for this suffering? That is something that I always feel when I looked at the infant. I mean when you need an important operation but you know that you’ll have a good life afterwards it’s completely different. When you have a lot of pain and suffering and it ends in nothing.” (PART. 5)
	“I think to use extensive medicine to keep the baby alive if you are quite sure that the quality of life of the baby is bad, I think it’s not a good thing. Nowadays in neonatology we can make nearly every baby survive. But sometimes if they have a big brain hemorrhage, we can do everything to keep the baby alive but we know that the quality of life for the baby will be quite poor, if we have all the parameters that really confirm that he’s going to have severe disability. And in my opinion it’s not a good thing to maintain life if the quality of life of the baby will be really poor.” (PART. 12)
Respect for parents’ autonomy	
	“But yet parents… it’s their decision. I find that parents are more important than me, ale’ of my opinion. If they don’t want that, it’s their right because I cannot promise that everything will be ok so if they really don’t want any option (meaning risk) that things will be bad then we cannot start.” (PART. 11)
	“Have I to convince the parents to resuscitate the baby? but if they don’t want am I right to decide for the parents? but it’s not me… I’m not the mother, it’s not my child, it’s not my family, it’s not me who all day will take care of this child and will live with all the consequences of prematurity. So yes it’s difficult, it’s very difficult. And when you see also the impact on the families and on the environment on the neurodevelopment of the child and how it’s important, it’s really important that the parents take part of the decision really.” (PART. 18)
	“(*Referring to a case in which parents asked non-resuscitation for a baby she believed might have survived)*
	*Int*. so despite your doubt about this case, would you still do the same?
	*Part*. yes yes. We also always tell our numbers of survival and handicap percentages, we tell the parents, and if they really don’t want to take the risk of having maybe 5 to 10% of having a little handicap, yes it’s their right. Yes, it is difficult but it’s their right. I still believe it was the right decision.” (PART. 18)
Parents’ interests	
	“(*Referring to a case in which resuscitated a baby at 22 weeks because it was important for the father’s mental health)* But in my point of view afterword I would do the same because I think that good perinatal medicine is not only treating newborn babies it’s treating a complete family. And I felt that the prematurity of this baby paid an important part of the health of this father and if we would have followed the mother’s opinion and didn’t do anything I don’t know how it would have ended with that man and with that relation. So I think that in my point of view medicine is a little bit more holistic and perinatal medicine has to focus on a family and not just on a child. And I knew I couldn’t save the child but by trying to save the child I treated the father.” (PART.2)
	“She died after 3 weeks I think so I think that the parents had 3 weeks of, I’m not saying it was all joy, but at least they had a bond with their baby, they saw it alive, they cuddled it, they did kangaroo care, they have pictures of her, they have all sort of memories… So I think yes I would do the same.” (PART. 14)
	“I think it’s a good practice because I think the survival chances at 24 and 25 weeks are like 60% in the group of the survival you will still have 40% of infants who will have developmental problems, major developmental problems. And for the parents, yeah parents are still quite young and they have the whole life to take care of the infants, so yeah I think it’s important that they are involved in the decision on whether to start or not because it’s their life and they have to take care of their infant.” (PART. 5)
Justice	
	“Economic factors, should they be important when we are talking about a life? I don’t think so. I don’t think so. It should not but you have it in mind. We have very preterm babies of addicted parents. These babies will go to guest families. So these are problems that can be solved. It’s not optimal but can be solved. […] So you can solve most social economic problems. We are a wealthy country so it should not be a problem. Sometimes it’s in your mind, can you switch it off? No, not really, but you can think in solutions.” (Part. 15)
	“You can do a lot of things with nature but sometime is too far. And when the studies say there is no good chance of having a good child and then when you see that it’s not only the child who has damage done but also the family, the parents, a lot of divorces, the brothers and sisters psychological problems with all attentions going to one child for the rest of the life. It’s too defused form everyone and for the economics of Belgium and the others to have all these handicapped child. It’s not that if you are handicapped…but if you know it in advance 99% that he would be handicapped I don’t see why you would do anything and give the parents another chance to have in a few months another pregnancy. It’s my opinion.” (PART. 13)

#### EPIs’ best interest

All participants perceived EPIs’ best interest as an extremely important principle and they often described themselves as being the EPI’s advocate or the defender. They said the goal of the decision-making should always be to identify the option corresponding to an EPI’s best interest. This principle consists of two elements: pursuit of the EPIs benefit, i.e., survival with good quality of life, and avoidance of unnecessary harm, i.e., therapeutic obstinacy.

Participants applied this principle in the described cases by doing a harm-benefit assessment to determine whether the harms associated with resuscitation were justifiable in light of the benefits gained. Clinical conditions and expected prognosis were the main elements of this assessment. However, participants were aware that there is more to the best interest of a child than mere clinical aspects. As one participant said “*We maintain life not vital parameters. I don’t want that only the monitoring is going well. I think that life is bigger than just your heartbeat or respiratory parameters.*” They all agreed that life in itself was not necessarily a benefit if the quality of life were extremely poor. Therefore, consideration of the probable long-term quality of life was an essential part of the assessment. Non-clinical elements like parents’ wishes were also considered very important. Participants also recognized that the best interest can be difficult to evaluate due to clinical uncertainty and that it can depend on subjective evaluation. Consequently, in the gray zone where the clinical uncertainty is high, parents’ wishes and parents’ interpretation of the best interest were considered more important than they were outside the gray zone.

#### Respect for parents’ autonomy

Respect for parents’ autonomy was the second most important principle that emerged from our analysis. Participants interpreted and weighted, or valued, this principle differently, depending on the EPI’s GA. Outside the gray zone, the decision was not really debated because the EPI’s best interest was clearer and because the guidelines explicitly indicated whether to resuscitate. Hence, respect for parents’ autonomy was described mainly as parental involvement. Participants said that they explained to parents the treatment plan for the EPI, but they did not really engage them in the resuscitation decision. However, participants still valued parents’ wishes and life history. They pointed out that it is important to give parents the opportunity to ask questions and express their fears and wishes. This helped them to build a relationship with parents and even to change the decision when necessary. Inside the gray zone, both resuscitation and non-resuscitation were considered ethically acceptable options. Participants actively engaged parents in the decision-making to understand which option was in the best interest of each EPI. The goal was always to reach a shared decision. In cases interviewees reported a disagreement, they felt that they had to respect the parents’ decision, and they generally followed it.

A few participants interpreted respect for parents’ autonomy differently. As reported by these participants, they informed parents of the clinical situation, asked their opinion about best treatment, and tried to understand parents’ point of view. They then used these insights to decide whether to resuscitate. This effectively meant that the physician made the ultimate decision, although it was heavily influenced by parents’ wishes.

#### Parents’ interests

According to participants, resuscitation decisions can have a severe impact on parents’ wellbeing and mental health. For example, parents of severely disabled children might face depression and divorce, whereas parents of non-resuscitated EPIs might feel guilt and regret. Because of this, participants believed that ethically sound decision-making respects not only EPIs’ best interest and parents’ autonomy, but also parents’ interests.

The attention participants paid to parents’ interests was evident at every stage of the described decision-making process. Participants remarked that it is very important to know parents well—not only their wishes but also their family history—and why they are asking for one or the other option. This background information was necessary to tailor the counselling and the decision-making to parents’ specific needs. They also explained that even when they had to refuse parents’ request, they still tried to maintain a positive dialogue with parents and to help them cope with the situation. In exceptional cases, participants even decided to resuscitate EPIs with very low chance of survival so that parents could feel a sense of relief that they did everything they could for their child.

#### Justice

The majority of participants reported reflecting on the ethical implications of considering parents’ socioeconomic status and gynecological history in the decision-making for resuscitation at birth. Participants agreed that, in theory, they should not make resuscitation decisions based on parents’ characteristics. In practice, however, they admitted that parents’ characteristics could be influential factors in some cases. For example, the majority of participants resuscitated—or were open to resuscitate—an EPI below GA 24 weeks in cases in which older parents had a history of IVF, miscarriages, stillbirths, or neonatal deaths. Some participants also admitted that below GA 24 weeks, it was difficult not to take into account particularly problematic familial situations (e.g., addictions, other children in foster care), because they knew that these factors have a documented impact on EPIs’ outcomes. In general, they were uncertain on whether to consider parents’ status and to what extent. Interviewees approached this issue differently. Some only considered parents’ status below GA 24 weeks for EPIs in critical condition. Some made conscious efforts to minimize the influence of parents’ socioeconomic status by trying to focus on practical solutions, e.g., helping parents to apply for social services specifically designed for their situation. Either way, they all agreed that parents’ status should never be the main reason to decline resuscitation.

In ethics justice can also refer to resource allocation. However, only one participant spontaneously mentioned resource allocation. She stated that extreme, severe disability can be unfairly difficult for the child, the siblings, the parents, and the state responsible for a citizens’ healthcare. However, she did not elaborate more about how justice as resource allocation might influence her decisions. When asked directly, all the other participants agreed that resource allocation should not be considered in the decision-making because Belgium is a high-income country with sufficient resources to offer aggressive treatment to every EPI who might benefit from it. They were also aware that in lower-income countries, economic considerations might inevitably contribute to decision-making; and they felt fortunate to be able to focus only on the best interests of children and their families without worrying about hospital resources.

### Ethical challenges

We identified three main ethical challenges experienced by participating neonatologists based on the cases they reported: (1) conflict between EPIs’ best interest and respect for parents’ autonomy, (2) limitations of the guidelines, and (3) dealing with clinical uncertainty. For illustrative quotes on ethical challenges, see Table 3.

**Table 3:** Illustrative quotes: Ethical challenges

Conflict between EPIs’ best interest and respect for parents’ autonomy	
	“It was very hard for us to perform resuscitation because we could see how our lack of possibilities. But on the other hand, for the father especially who came here and assisted to resuscitation, it was maybe something he really needed for seeing that we really tried everything and maybe if we wouldn’t have done it, it could have stayed like it was like we didn’t fight enough. From the parents’ point of view I think it was necessary and it was probably what they needed. On the other end there was also the suffering of the fetus or newborn so that makes it more difficult for us.” (PART. 3)
	“In whose interest do you need to make your decision? it’s a question that comes up here every day eh!? To do good for the parents sometime the children have to suffer. And we know that the prognosis is not good or that whether the baby will die now or in a month it will just be a month less suffering. But the parents need to live with it and they need to be at ease with it. That’s for me one of the main ethical questions: whose interest do you need to follow? Do we need to be the advocate of the child but the parents are sitting in front of you with their sorrows and their aspirations and their hopes so yeah that’s difficult.” (PART. 6)
	“I had the feeling that a potentially very good baby didn’t have a chance because some people really have the idea that prematures are weak and they have handicap. Sometimes common sense is like “oaah!” If you also tell someone that you are a neonatologist they always think you’re only deliver handicapped babies but that’s not true at all! And still that is in many people ideas. And I had the idea that those parents were also people that had that idea, like a baby at 24 weeks never can be ok, but that’s not true! And there I have ethical difficulties with because they were so much convinced of their own believes that there were not always facts but yeah I cannot force them! it’s their baby! who am I?! but as a doctor that’s more difficult”. (PART. 11)
	“I find it very difficult that she was refusing antenatal steroids, ok that’s her choice, but she cannot expect the day that she is delivering to change her mind! She did and so now we have lung problems! That’s very difficult. For me when you choose not to give active care and then you change it and then he is not in an optimal situation the baby is the one who suffered now because she didn’t want to give it. We wanted it, she didn’t, then she changed her mind, now he is the one who has the lungs problems. So he is the one who now has the problem he is.” (PART. 14)
Limitations of the guidelines	
	“If there are technical limits that make it impossible, then I have something like “ok that’s the limit, it’s a technical one”. If it’s technical for me is easier. If the limit is just a protocol, that’s more difficult
	*Int*. and how do you deal with a protocol limit?
	*Part*. well, that’s like the 24 weeks issue. The protocol says no below 24. If parents say “we would like you to start” and I would start, then I’d go against the protocol.” (Part. 7)
	“I always agree if we are sure that the baby is not having a digniful life that we should stop. So in that way I’m not conservative I think, but to start something? I think we should start more. I think if it’s not working if the baby has big problems we can always do something to end the life anyway. So I’m not scared of trying more and I think that we should do it and we should let the parents decide. We should say from 25 weeks we should always do active care, 24 weeks. For me I just think that 26 weeks it’s too late. I don’t know if you have seen many preterm babies but there are many babies that at 25 weeks start crying and it’s hard not to do anything even for caretakers. We are professionals we are trained to help we are not trained to let die.” (Part. 14)
	“ (*Referring to a case in which parents refused resuscitation for 25 weeker in good condition*) You’re almost convinced that these babies could survive and could have a very good life, qualitative life. So… and there’s also some anger that we have this guideline and we discuss the guideline with the parents and the parents choose for what you not expect. Then you think “why do we have this guideline?” because this guideline will work maybe for the whole group of 25 weekers but there’s much of diversity and I think these babies were much better than the median group. So it’s a little bit of frustration that you cannot do what you think it’s best for the babies.” (PART. 15)
Dealing with clinical uncertainty	
	“I have no arguments to say that the prognosis will be bad but I have no arguments to say that we will perfect. […] (referring to the colleagues who complained because she resuscitated an EPI of 22 weeks) If they want the perfect patient, the perfect baby that will go home, then they shouldn’t probably work in a neonatal centre. You know you need to be… as a doctor you have to accept that you’re not controlling everything. We always say that we are the defender of the child so if there is a chance of good outcome we should give that chance to the par-the child. If you want certainty you probably you shouldn’t resuscitate any of them.” (PART. 4)
	“Well one thing and that’s really also an ethical issue and I think it’s a difficult issue. That we know that if we treat 23 weeker we are not so experienced with that because the numbers are very small or almost zero. We don’t know yet how to treat them. so it’s maybe kinda of an experiment you could say. And it is really different from what we are used to in 25/26 weekers, it really is! So there are unexpected things we didn’t see in other babies. It’s an evolution but it is an experiment! So you move from the limit of viability and you experience again new things. So you are learning. For instance we used to disinfect with alcohol, you cannot do that with 22 or 23 weeks they get burns. Their skin is not ready for that! So you learn and sometime yeah it is an experiment and they don’t get yet the optimal treatment, we know that! But by learning this, the babies of 24/25 weeks are treated much much better because you we learn from these babies and our treatment for these babies is getting better and better and better. And that’s really an ethical issue: should we experiment in these babies to improve the care for a little bit older babies? When we look back 10 years ago we experimented on these babies and these babies were on profit of that so we are moving this way. So that’s an issue.” (PART. 15)
	“(*comparing two cases*) it was easier in that case because even though it was a difficult situation the possibilities of having good outcomes were not very high but were there, while in the first case we were quite sure that our
	techniques and our resuscitation would not lead to good results. So in the first case we didn’t have a lot of.. we were quite sure that the probabilities of resuscitating was really really low; in the other case there was more doubt so we felt at ease to doing.” (PART. 3)

#### Conflict between EPIs’ best interest and respect for parents’ autonomy

The main ethical challenge emerged from cases described by participants was a conflict between EPIs’ best interest and parents’ autonomy. All the described cases shared the same main characteristics with regard to this particular challenge. All were cases in the gray zone, where the guidelines accept both resuscitation and non-resuscitation and recommend to decide through shared decision-making. From the interviews we noticed that many participants gave a different interpretation of shared decision-making. They felt that the ultimate decision is of the parents and that their role was mainly to enable parents to make such a decision and to respect it. Thus, participants felt that they had to accept parents’ requests, despite believing such a request was against the best interest of the EPI. This happened when parents insisted on initiating resuscitation at birth for an infant in critical condition, or when parents asked that their EPI in good condition *not* be resuscitated. For many participants this was a source of moral doubt, that is, a sense of uncertainty of which action is morally justified. On the one hand, they told us they should be the child’s advocate, and they felt frustrated that they could not act like it. On the other hand, they still valued parents’ autonomy, and they questioned whether overruling the parents’ decision would have really been the right choice. Moreover, despite the internal discord, participants often showed that they understood why parents were making such a request. This realization made it even more difficult to consider the possibility of overruling them. In rare instances, moral doubt turned into moral distress. Two scenarios led to such moral distress. First, when a resuscitated EPI died after months in the NICU and after undergoing multiple painful interventions. Here, participants felt that they had actively harmed the child and that the harm was pointless since the EPI died as predicted. Second, when a non-resuscitated EPI in very good condition at birth took hours, or even days, to die. Participants interpreted this slow death as proof that the EPI might have survived if resuscitated.

Actually, our analysis indicated that these kinds of conflicts between principles could occur also for EPIs born outside the gray zone. However, in these cases, participants felt justified in refusing parents’ requests, because their decision was well within the guidelines’ indications. Therefore, these situations were quickly resolved without generating long-lasting moral dilemmas or moral distress.

#### Limitations of the guidelines

Another important ethical challenge appeared in reported cases where participants perceived limitations in the guidelines. The majority of participants explained that they used the regional or unit guidelines to guide the decision-making. For them, guidelines were based on best interest and autonomy considerations and, therefore, they were a useful and appropriate tool to guide the decision-making. However, because of the clinical and ethical complexity of EPI resuscitation at birth, they felt that the guidelines could not cover every single situation. For situations not addressed by the guidelines, the guidelines actually created an untenable imbalance between principles. In these situations, participants felt that the guidelines put more weight on some principles but overlooked others. This made it difficult for participants to identify the best option and to act accordingly.

For example, for EPIs born at GA 25 weeks, the majority of Belgian guidelines recommend allowing parents to decide whether to resuscitate. However, some participants believed that the overall outcomes were sufficiently favorable to warrant a resuscitation attempt, and not doing so meant failing to protect the best interest of the EPI. Similarly, at GA 23 weeks, most guidelines recommend non-resuscitation, but some participants believed that sometimes this could infringe on parents’ autonomy. Therefore, they proposed lowering the gray zone to GA 23–24 weeks. According to them, this would allow neonatologists to resuscitate EPIs in good condition at GA 25 weeks and to protect the child’s best interest. It would also allow them to offer resuscitation at GA 23 weeks. They explained that, at present, they do not offer resuscitation at birth for EPIs born at this age, although they are open to perform it if parents actively request it. For others, the main problem was not the specific GA threshold, but instead their unit’s inflexible interpretation of the thresholds, which were followed strictly. These participants believed that more flexible thresholds would allow them to balance the principles on a case-by-case manner, and this would help them decide which one should be weighed more in a specific case. Evidently, guidelines are always prone to change and also in Belgium there will be adaptations in the future.

#### Dealing with clinical uncertainty

The last main ethical challenge described by neonatologists in our study related to uncertainty. Interviewees explained that deciding whether to resuscitate EPIs at birth meant dealing with clinical uncertainty. Determining the chances of survival and quality of life for each EPI is challenging. In participants’ words: “… these infants surprise you.” Many participants mentioned cases in which an EPI in critical condition survived and had a good quality of life. In other cases, an EPI in good condition died due to a deleterious postnatal event. In participants’ cases, clinical and moral uncertainty became linked: As clinical uncertainty increased, it became more difficult to decide whether resuscitation or non-resuscitation was the best option for that EPI.

Participants acknowledged that clinical uncertainty is not specific to EPI resuscitation but is intrinsic to neonatology. Indeed, we noticed that, compared to the previously discussed challenges, participants seemed more prepared to be confronted with clinical uncertainty. In their words, to be a neonatologist, one must accept that one cannot control everything and that there will always be a certain level of uncertainty associated with their decision, whatever that is.

In some of the described cases, clinical uncertainty seemed to have simplified the decision-making somewhat. The guidelines let parents make resuscitation decisions in the gray zone due to the high clinical uncertainty associated with EPIs born in this period. When faced with mounting clinical uncertainty, it was easier for participants to accept parents’ requests compared to situations in which an EPI was born in the gray zone, the prognosis was clearer, and they felt that parents’ requests were against the best interest of the EPI. From the cases neonatologists described we observed that when the prognosis was highly uncertain they had no elements to say that parents’ request was against the interest of the EPI, even if maybe they personally would have chosen a different option. Hence, it was somehow easier to comply with parents’ request because their objection was more personal than professional and they believed that purely personal opinions were not enough to interfere with parents’ autonomy in the grey zone. When the prognosis was more certain and parents’ request was against what they believed was in the best interest of the EPI based on medical data, than they felt a conflict between EPIs’ best interest and parents’ autonomy as described previously.

Some participants also discussed uncertainty in terms of treatment, rather than only outcomes. They explained that they did not have much experience treating EPIs at GA 22–23 weeks, since there are so few cases. Of these, only a small number of infants are resuscitated. As their experience was mainly anecdotal, they could not guarantee that the same treatments given to older EPIs would also benefit younger EPIs. They also agreed that by resuscitating more EPIs at GA 22–23 weeks, they would gain more knowledge and be able to improve treatment not only for these infants but also for older EPIs. However, they remarked that gaining knowledge should never be the primary reason to resuscitate.

### Strategies to deal with ethical challenges

Despite the diversity of participants’ experiences, all interviewed neonatologists described two kinds of strategies to deal with these ethical challenges. They can be broadly characterized as “setting limits” and “trial of treatment.” Illustrative quotes on the strategies they used to deal with ethical challenges are presented in Table 4.

**Table 4:** Illustrative quotes: Strategies to deal with ethical challenges

Setting limits	
	“Afterward, we asked ourselves “was it right to resuscitate or not? We should have been maybe more strict with the parents considering that we have seen the technical difficulties and we were a little bit concerned that was it acharnement or not?” So there was a little bit of discussion about it but finally, especially seen the fact that for the parents was very very important as well to at least try to do something. And at the same time we still put some limits. For example we won’t intubate, perfusion, we won’t go to for example giving cardiotropic drugs. So finally after discussing it all together we consider that it was very tough for us but not necessarily a bad decision. Especially that particular situation: not having the whole information, not really the whole proof about the medical information, not having the time to waiting for the baby. So it was a complicate situation, but considering all the limits there were, maybe it was the good choice to do.” (PART. 3)
	*“Int*. the whole team agreed to take care of the baby, why?
	*Part*. because the parents clearly told us their opinion. They really wanted to try. They knew the risks and it was just one day before 24 weeks. I don’t like the expression” precious pregnancy” but they have gone through so many dramatic situations and the parents knew that it could have been difficult for the baby but they really wanted us to do everything we could do. We told the parents “we can try, but if it’s too heavy for the baby, if it’s too difficult, if he is too much premature we will stop resuscitation, we will stop intensive care.” (PART. 12)
	*“Int*. and in the case in which the baby would survive majorly impaired, if the parents want resuscitation would you provide it?
	*Part*. we can do that yeah. But sometime there is no time to discuss with the parents and then you do but you also keep in mind what is good for the baby so there will be limits. If we have to give thorax compression and adrenaline and the baby is not responding I would be the first and very quick to say: it doesn’t work I’m sorry but your baby is not going to survive”. Sometime you have to do something just to give the parents the feeling that we gave the baby really a chance.” (PART. 15)
Trial of treatment	
	“Stabilizing a baby after birth and then making a decision based on the first 2/3 days of life… I don’t think it’s fundamentally different, I just think we have more elements to make a more wise decision […] Deciding to let him die or accompany him or her to die in a painless way 1 h after birth or 3 h after birth it doesn’t make any difference. I mean philosophically speaking I don’t see a difference. Instead I personally feel more comfortable if I verify that there are things that are compromise his survival anyway versus not. I think this really makes a difference because if he really has a brain hemorrhage I feel much more positive I feel like I make a decision based on facts rather than on speculation because the gestational age number is a bit of speculation whereas brain hemorrhage in your head is a fact.” (PART. 16)
	*“(Explaining why she prefers trial of treatment than withholding treatment)* [withdrawal] It’s better I think, but it’s harder for us as humans. But it’s better for the parents and they really know we did everything but it was impossible. Otherwise they will have questions “what if the baby would have had all the chances?! Would it be different? Maybe we would have had a healthy baby?” and then they start thinking at that later maybe? But then they really know how it was and they could decide. The parents have a big decision ere- with good advice, medical advice- but eventually the parents decide.” (PART. 18)
	*“(Referring to a case in which initially parents disagreed on whether to start treatment. I asked what if parents did not solve their disagreements)* I think I would give active treatment. With all the clear explanation also that if there are huge problems afterward we are not going to do absurd things. It must be reasonable. If we see that the baby will have severe complication, that will have severe handicap after in life we will have to discuss again and maybe then for the mother it would have to turned. But when there is doubt I think, I don’t know if it’s right in English, but we have to give the advantage of the doubt.” (PART. 5)

#### Setting limits

As described by participants, this strategy consists of restricting parents’ requests to varying degrees (i.e., what the physician would/would not attempt) to promote the EPI’s best interest. Depending on the situation, different participants used different limits, and they restricted parents’ requests to a more or lesser extent. Participants employed this strategy mainly when they perceived an actual or potential conflict between principles.

The easiest reported way to set limits was referring to the guidelines. For example, if parents requested non-resuscitation above GA 25 weeks, participants explained that they had a professional limit vis-à-vis the guideline and that they would not accept a non-resuscitation request at this GA. However, they would still try to understand why parents asked non-resuscitation so that they could address parents’ worries. Participants said these parents were often afraid of futile treatment, disability, or they thought the situation was worse than it actually was. In these cases, participants would reassure parents that if the infant did not respond well to treatment after admission in intensive care, they would discuss with them the possibility of withdrawing treatment. In this way, they protected the EPI’s best interest as well as respected parents’ interests and autonomy.

Participants explained that they would make exceptions to the guidelines, but they would still set limits in these exceptions. At the time of the interview, most participants agreed to resuscitate even below the recommended threshold to respect parents’ autonomy and to preserve their wellbeing. However, to protect the EPI from therapeutic obstinacy, they all set limits on the extent of care they would provide. For example, in this scenario, they agreed to initiate resuscitation, but they made it clear to parents that they would not provide CPR or adrenaline.

Many participants tried to set the same limits also in the gray zone when they disagreed with parents’ request. However, due to clinical and moral uncertainty, participants often struggled to determine what limits to set and even whether limiting parents’ request was the right thing to do. In some cases, setting limits was not possible due to lack of time for appropriate counselling, or because parents persisted in their request. These situations were often the ones in which we observed long-lasting moral distress. Moreover, we observed that these kinds of cases seemed more common in units where guidelines were rigidly interpreted. Here, participants felt it to be unethical to go against guidelines if parents did not agree with it.

#### Trial of treatment

The second strategy described to deal with ethically challenging situations was “trial of treatment.” This second strategy consisted of attempting resuscitation at birth with the understanding that if the EPI was not responding well or developed a severe postnatal event in NICU, physicians would withdraw treatments. This strategy gave participants a sort of moral relief. Participants explained that it is difficult to obtain a clear and sure prognosis before or even at birth. Therefore, resuscitating and seeing how the EPI reacted allowed the participants to “give a chance” to the EPI and allowed them to take time to gather better information on possible outcomes. Moreover, parents had the opportunity to spend some time with their child, and they could see that there was nothing else to do. This, in turn, might reduce the risk of feeling regret. Some participants admitted that trial of treatment is not the best option in every situation. However, because of the many advantages, this strategy seemed to be the preferred option in the majority of cases, with some participants advocating for more resuscitation attempts even at the earliest GAs.

## Discussion

Two ethical principles were central in neonatologists’ ethical reasoning regarding resuscitation/non-resuscitation decisions at birth: the EPIs’ best interest and respect for parents’ autonomy. Participant neonatologists considered and aimed to strike a balance between these two principles in almost every case. This was not surprising, as EPIs’ best interest and respect for parents’ autonomy commonly appear in the ethical literature as justifications for both resuscitation and non-resuscitation of EPIs [11]. Furthermore, these principles are also central in Belgian guidelines for resuscitation of EPIs [31]. This could explain why, among all the ethical principles discussed in the interviews, these two were consistently the most important for participants in their decision-making. Guidelines also had a relevant place in participants’ ethical reasoning. Indeed, most participants used them to some degree to make decisions at birth. Guidelines provided general rules, principles, and advice as well as something to “anchor” participants’ decision-making in such a complex and emotionally sensitive area. A related study on the impact of institutional ethics policies on clinicians’ dealing with euthanasia requests in Belgium found similar results [35]. Euthanasia policies influenced clinicians’ practices and perspectives on euthanasia. Moreover, policies guided clinicians through the euthanasia process and supported them in such complex and sensitive decision-making.

The importance placed on EPIs’ best interest and respect for parents’ autonomy can explain why conflicts between these principles were perceived as the most difficult ethical challenge. Participants felt compelled to advocate for the EPI. They also acknowledged that there is always a certain degree of uncertainty regarding outcomes, making it difficult to overrule parents. Participants’ reflections showed that, although ethicists agree that neonatologists should theoretically refuse parents’ requests that are clearly against the EPI’s best interest [36–45], this can be difficult to do in practice. Our analyses revealed that conflicts between principles could be even more acute in the GA gray zone. This might be because outside the gray zone participants had more resources to approach the situation in an ethically satisfying way, whereas inside the gray zone, there was an added layer of complexity introduced by the guidelines. Most Belgian guidelines assigned parents as the main decision-makers inside the gray zone because of the high clinical uncertainty that characterizes births in these weeks. However, not every case in the gray zone will be highly uncertain; sometimes there are favorable indicators about the EPI’s likelihood of survival and quality of life. Participants tended to accept parents’ requests, even when they believed that such requests were clearly against the best interest of the EPI. They acquiesced because they felt that they did not have the authority to override the guideline, and hence parents’ requests. Although these situations are rare, they often resulted in moral distress, especially in units where guidelines were interpreted rigidly. Participants felt guilty and frustrated for not being able to advocate for the child; some gave very emotional and distressed accounts, even years after the event. Thorne et al. described similar reactions in a study on moral distress response patterns in NICUs [46].

Studies on moral distress of healthcare professionals in NICUs found that, although moral distress can arise in any complex decision-making, it is often associated with treatment decisions for EPIs [47, 48]. Institutional structures, in particular unit culture and inadequate guidelines, were often reported as one of the causes of moral distress [46–48]. Interestingly, studies found moral distress much more prevalent in cases in which practitioners felt they were “doing too much” compared to cases in which parents opted for palliative care [47, 48]. We observed that moral distress arose from both resuscitation and non-resuscitation situations so not only when they felt they were doing “too much” but also when they felt they were “not doing enough”. This outcome might be a result of the specific Belgian context. While international guidelines advise physicians to start resuscitation at GA 25 weeks, most regional and unit guidelines in Belgium place the gray zone at GA 24–25 weeks [49]. This means that parents can opt for non-resuscitation even at GA 25 weeks. However, many participants felt that the guidelines were too conservative. They believed that at 25 weeks the outcomes are good enough to warrant a trial of treatment, and they felt that non-resuscitation would go against the best interests of EPIs. Finally, although all NICU healthcare providers experience moral distress, most studies still focus on nurses’ experiences [47]. The field needs to better understand how neonatologists experience moral distress and to identify its causes and consequences. Such knowledge is imperative in order to support neonatologists in their decision-making and help them to efficiently handle moral distress.

Finally, our analyses revealed that participants were more interested in the impact that resuscitation decisions have on EPIs and their family rather than the impact that they might have on society at large. This was particularly evident when we looked at how participants discussed justice in the interviews. Only one participant mentioned resource allocation spontaneously, and when asked directly, everyone agreed that it should not influence their decision-making. This unanimity could be because Belgium is a high-income country with a well-financed and organized public health system, meaning that doctors and parents are not constrained by the cost of care. This finding is almost identical to what we found in the ethical literature: Few articles mentioned justice, and they all agreed that it should not be considered in decision-making [11]. However, these studies, like ours, were from high-income western countries; thus, they are hardly generalizable on a global level. In fact, studies originating from lower-income countries [16, 50–54] described situations in which neonatologists had to deal with lack of hospital resources (e.g. lack of sufficient ventilators) or parents’ inability to pay the hospital bill. These situations can affect the care provided. For example, a study from the Philippines found that if parents are unable to afford renting a ventilator, physicians would attempt hand ventilation or they would try taking of support infants on low ventilation settings hoping they will not need it anymore [53]. Lack of resources in some instances can lead to consider hospital or family resources in the decision-making [16, 50–52, 54]. A study from Lebanon found that hospital resources is the second most relevant factor in resuscitation decisions after the infant’s prognosis. Similarly, a participant in an Indian study explained that he would not attempt resuscitation below 28 weeks if parents were not able to afford it. [51] However, in all these studies we see attempts to overcome the limited resources to provide the best care possible to the patients. This suggests that neonatologists in lower-income countries still value EPIs’ best interest more than justice but, due to unfavorable circumstances, they have to take justice into account. This is even more evident in a study investigating Chinese neonatologists’ attitudes toward resuscitation of EPIs and the impact of cost of care on their attitudes. Ma et al. found that the cost of care was a primary factor in deciding whether to resuscitate [16]. Interestingly, 39.7% of respondents in this study indicated that they would seek financial aid to support families who cannot afford care. Similarly, some our participants indicated that they would try to help parents with low socioeconomic status to find the necessary resources for post-discharge EPI care.

However, justice as an ethical principle can also refer to avoiding discrimination. To this regard, participants in our study were particularly concerned with discrimination related to parents’ socio-economic status. There is a documented relationship between parents’ socioeconomic status and EPIs’ neurodevelopmental outcomes [55–57]. Thus, participants asked if and to what extent they are allowed to consider it in the decision-making. It is our impression that many participants were hesitant to discuss this topic. This hesitancy might have limited our understanding of the real impact of justice on decision-making.

### Strengths and limitations

Our results originated from a fairly large sample of neonatologists. We interviewed 20 neonatologists (i.e., 18% of the total population of neonatologists in Belgium) working in all three Belgian regions (Flanders, Brussels, and Wallonia). We included 10 out of the 19 Belgian hospitals with a NICU. Participants and hospitals present a good variety in terms of relevant characteristics. However, our sample is rather homogenous in terms of ethnicity, gender, and religious affiliation, which might have limited the generalizability of the results. Similarly, there is an underrepresentation of NICUs with a larger number of beds (only one NICU had more than 35 beds). This might have again affected the results and their generalizability as NICUs with more beds normally treat more cases and are more experienced in this type of decision-making. Moreover, neither the participants nor the interviewer are native-English speakers. This might have hindered participants’ accounts of their experiences, feelings, and thoughts, especially regarding distressing topics like moral distress or the impact of parents’ socioeconomic status. Finally, we asked participants to describe real past cases to avoid discussing hypothetical scenarios and to have a better understanding of real practice ethical decision-making. However, these are still a posteriori* reported* cases that cannot give a complete and comprehensive description of clinical practice.

## Conclusions

Our results showed that in making resuscitation decisions for EPIs, participants always tried to balance the EPI’s best interest and respect for parents’ autonomy. Because of this weighing, they perceived it to be particularly challenging when dealing with cases in which these two principles clashed. When participants could successfully set limits to parents’ requests without overriding them entirely, they felt satisfaction because they could protect the EPI’s best interest while respecting parents’ autonomy. However, when setting limits was not possible, participants often developed moral distress. This suggests that we need to better understand neonatologists’ moral distress in EPI resuscitation decisions to develop better strategies that can help neonatologists cope.

## Data Availability

The datasets generated and/or analysed during the current study are not publicly available to protect the privacy of participants as well as other persons involved in the discussed cases but are available from the corresponding author on reasonable request.

## Abbreviations

EPI: Extremely premature infants
GA: Gestational age
NICU: Neonatal intensive care unit
QUAGOL: Qualitative analysis guide of Leuven
